# Tailoring Microstructure and Properties of CoCrNiAlTiNb High-Entropy Alloy Coatings via Laser Power Control During Laser Cladding

**DOI:** 10.3390/ma19010005

**Published:** 2025-12-19

**Authors:** Zhe Zhang, Yue Yu, Xiaoming Chen, Li Fu, Xin Wei, Wenyuan Zhang, Zhao Dong, Mingming Wang, Tuo Wang, Xidong Hui

**Affiliations:** 1 School of Materials Science and Engineering, Xinjiang University, Urumqi 830017, China; 2 Water Machinery and Remanufacturing Technology Engineering Laboratory of Zhejiang Province, Hangzhou River Mechanical and Electrical Equipment Engineering Co., Ltd., Hangzhou 310024, China; 3 Key Laboratory of Surface Engineering of Equipment for Hydraulic Engineering of Zhejiang Province, Standard and Quality Control Research Institute, Ministry of Water Resources, Hangzhou 310024, China; 4 Yellow River Water Resources and Hydropower Development Group Co., Ltd., Zhengzhou 450045, China; 5 State Key Laboratory for Advanced Metals and Materials, University of Science and Technology Beijing, Beijing 100083, China; dongzhao_ustb@163.com; 6 China Yajiang Group Co., Ltd., Nyingchi 860013, China; 15638296760@163.com

**Keywords:** laser cladding, high-entropy alloy coating, laser power, cavitation erosion

## Abstract

To enhance the operational damage resistance of hydraulic machinery, this study employed laser cladding technology to fabricate a Co_37.4_Cr_30_Ni_20_Al_5_Ti_5_Nb_2.6_ high-entropy alloy coating on 04Cr13Ni5Mo substrate. The influence of laser power on the microstructure and properties of the coating was systematically investigated. Based on preliminary research, the friction-wear performance and cavitation erosion behavior of the coatings prepared at 3000 W, 3200 W, and 3400 W were specifically examined. Results indicate that as the laser power increased from 3000 W to 3400 W, the microhardness of the coating gradually decreased from 345.3 HV_0.2_. At 3000 W, the precipitation of trace strengthening phases significantly enhanced the mechanical properties. In wear tests under a 20 N load for 30 min, the wear rate of the coating prepared at 3000 W was 1.41 × 10^−4^ mm^3^/(N·m), which is 13.5% lower than that of the 3200 W coating (1.63 × 10^−4^ mm^3^/(N·m)) and 16.07% higher in wear resistance compared to the substrate. Cavitation erosion tests revealed that after 20 h of ultrasonic vibration, the mass loss of the 3000 W coating was only 2.35 mg, representing an 88.89% reduction compared to the substrate (21.15 mg), and significantly lower than that of the 3200 W (4.57 mg) and 3400 W (3.85 mg) coatings. This study demonstrates that precise control of laser power can effectively optimize the cavitation erosion resistance of high-entropy alloy coatings, providing technical support for their application in harsh environments.

## 1. Introduction

Cavitation is the premise of protection in the operation of water turbines. Cavitation occurs when the local pressure of the liquid is lower than the saturated vapor pressure during the operation of the flow component in the liquid [1]. Bubbles are generated, and a large number of bubbles are generated on the surface of the flow component and collapse, resulting in ultra-high-pressure stress shock waves and high-speed microjets, which repeatedly impact the material surface and cause damage. This is the phenomenon of cavitation [2]. Although cavitation collapse occurs very briefly, its destructive effect on the material is enormous. Cavitation severely compromises the normal operation of flow components, shortens service life, and creates safety hazards, making it a key concern in industries such as hydraulic engineering, aerospace, petrochemicals, shipbuilding, and biomedical applications. During the operation of high-speed propellers, hydrofoils, pumps, valves, and other flow components, severe cavitation damage often occurs [3,4]. The key to addressing cavitation effects lies in creating a dense, cavitation-resistant coating on material surfaces. However, in practical applications such as marine engineering and hydropower projects, cavitation and sediment abrasion often occur simultaneously, accelerating material degradation. When designing protective coatings for extreme fluid dynamics environments, it is crucial to establish a synergistic protection system that combines superior cavitation resistance with better wear resistance capabilities.

High-entropy alloys (HEAs) represent a paradigm shift in materials design, departing from the traditional approach of using a single principal element [5]. Through multicomponent homogenization design, they transform material properties from being dominated by a single element to achieving synergistic effects among multiple elements, significantly enhancing the performance of alloy coatings. The coordinated interaction between complex compositions and multiscale structures, along with structural regulation at different scales, enables HEAs to activate complementary plasticity mechanisms during deformation. This ultimately achieves a synergistic enhancement of high strength and high ductility. The rapid cooling and heating characteristics of laser cladding technology not only refine microstructures but also prevent compositional segregation, enhancing the solubility limits of elements in the coating. Compared with the techniques of thermal spraying and thin film deposition, the laser cladding has high bonding strength, dense and uniform microstructure, and no obvious porosity; work efficiency is greatly improved. Furthermore, the high-entropy effect inhibits the nucleation and growth of equilibrium phases, particularly brittle intermetallic compounds, while promoting the formation of metastable solid solutions. These synergistic effects significantly boost the demand for laser-clad high-entropy alloy coatings [6,7]. To achieve widespread industrial application of high-quality coatings, optimizing laser cladding process parameters is crucial. Among key parameters, including laser power, scanning speed, powder feeding rate, overlap ratio, and shielding gas, laser power stands out as the most critical factor. This prominence stems from its direct control over energy input, which fundamentally determines microstructural changes in the material. Ren et al. [8] conducted a comprehensive investigation into the microstructure and wear resistance of near-atomic FeCoNiCuAl high-entropy alloy coatings under varying laser energy densities. Experimental results demonstrated that columnar crystals growing perpendicularly to the molten pool boundary were formed under optimal laser power conditions. The microstructure composition, particularly the specific orientation of grain morphology, significantly enhances the wear performance of the coating. Zhang et al. [9] developed FeNiCoCrTi0.5 coatings using laser cladding technology with varying process parameters, investigating their effects on microstructural properties. Experimental results demonstrated that as power increased, dendritic and columnar structures in the coating significantly expanded in size. The coating exhibited optimal wear resistance when thermal input was maintained at 72.22 J/mm^−2^. Zhao et al. [10] developed a CoCrFeNiMn high-entropy alloy coating on 304 stainless steel using laser cladding technology, investigating how laser energy density affects macroscopic residual stresses, crystallographic characteristics, microhardness, and cavitation resistance in the alloy coating. Experimental findings showed that the elements of coating and substrate interdiffuse to form the transition zone of composition. During solidification of the molten pool, Cr, Mo, and other elements are enriched in the interdendrite or grain boundary. When the laser energy density was reduced from 45 J/mm^2^ to 36 J/mm^2^, pore defects and microstructural coarsening in the coating were effectively prevented. Cavitation tests conducted after 10 h revealed that the coating exhibited a significantly lower average depth erosion rate compared to the 304 stainless steel substrate. The study demonstrated that the superior cavitation resistance of the high-entropy alloy coating stems from its unique grain structure, which effectively suppresses microplastic deformation. Furthermore, the passivation film on the coating surface maintained high integrity and continuity even under prolonged cavitation exposure. Yu et al. [11] developed an FeCoNiMnAl high-entropy alloy coating on 304 stainless steel using laser cladding technology. The study investigated the effects of laser energy density on the microstructure and properties of the FeCoNiMnAl alloy coating. Experimental results demonstrated that when the laser energy density was reduced from 40 J/mm^2^ to 24 J/mm^2^, the phase composition of the FeCoNiMnAl alloy coating remained unchanged, maintaining a single body-centered cubic solid solution structure. The average grain size of the coating decreased with reduced laser energy density. At 28 J/mm^2^, the coating exhibited excellent forming quality and superior overall performance, with a specific wear rate of only 8.2% compared to the 304 stainless steel substrate. Additionally, under this laser energy density, the sample demonstrated the lowest average erosion rate after 10 h of cavitation exposure, significantly lower than that of the base material. These findings indicate that selecting appropriate laser cladding process parameters is crucial for enhancing coating performance.

However, research on the cavitation erosion resistance of laser-cladded high-entropy alloy coatings remains scarce. Moreover, existing studies have not specifically addressed the Co_37.4_Cr_30_Ni_20_Al_5_Ti_5_Nb_2.6_ high-entropy alloy coating, nor have they focused on optimizing its cladding process to enhance cavitation erosion and wear resistance. In this study, Co_37.4_Cr_30_Ni_20_Al_5_Ti_5_Nb_2.6_ high-entropy alloy coatings are fabricated on conventional turbine stainless steel surfaces using laser cladding technology with varying parameter configurations. This research systematically investigates the effects of laser power on the macroscopic morphology, material phases, microstructure, hardness, wear resistance, and cavitation resistance of the Co_37.4_Cr_30_Ni_20_Al_5_Ti_5_Nb_2.6_ coatings. This research aims to solve the problem of material degradation of turbine components under harsh conditions through process control. The ultimate goal is to obtain coating materials with high hardness, excellent wear resistance, and cavitation resistance, thereby reducing material loss and extending service life.

## 2. Materials and Experimental

### 2.1. Experimental Materials and Laser Cladding Process

The stainless steel 04Cr13Ni5Mo was selected as the base material, with composition details shown in Table 1. The base material was cut to a size of 50 mm × 100 mm × 30 mm. Prior to laser cladding, surface rust was removed using 80–600 grit sandpaper in sequence, followed by alcohol cleaning and drying. High-purity (over 99.5 wt%) and uniformly sized (≤50 μm) powders of Co, Cr, Ni, Al, Ti, and Nb were used as raw materials for preparing Co37.4Cr30Ni20Al5Ti5Nb2.6 coatings. Prior to the experiment, the powder was baked in an 80 °C drying oven for 1–2 h. The homogenized alloy powder mixture was then loaded into a powder feeder to prepare the laser cladding coating. Based on preliminary experience, the hardness is unstable and the forming quality is poor when the power is lower than 3000 W, which leads to poor performance of the experiment, so it was not taken as the research object. The following cladding process was selected for sample preparation and performance testing, as detailed in Table 1.

### 2.2. Performance Testing

After the coating was fused, the coated sample was ground on a grinding machine and cut into 10 mm × 10 mm × 4 mm specimens using molybdenum wire cutting. Metallographic samples were collected along the cross-section of the Co_37.4_Cr_30_Ni_20_Al_5_Ti_5_Nb_2.6_ coating. The cross-section was treated with aqua regia for corrosion. The cross-sectional morphology of the Co_37.4_Cr_30_Ni_20_Al_5_Ti_5_Nb_2.6_ coating was observed under an optical microscope. The core degradation mechanism of the matrix material is the sensitization phenomenon induced by laser thermal cycling in the heat-affected zone, that is, Cr_23_C_6_ carbide precipitates at the grain boundary and forms a chromium-depleted zone, which leads to the transformation of the microstructure and residual stress, and destroys the ability of stainless steel to form the passive film of Cr_2_O_3_ spontaneously, thus leading to intergranular corrosion. The microstructure of the coating was characterized by field emission scanning electron microscopy (FE-SEM, ZEISS Supra 55, Baden-Würburg, Germany), with the accompanying energy dispersive spectrometer (EDS, Zeiss Merlin Compact, Baden-Würburg, Germany) employed for structural characterization. X-ray diffraction (XRD, X′Pert PRO, Panalytical, The Netherlands) was employed to analyze the phase structure. The microhardness tester (HXD-1000IMGCD, Taiming, Shanghai, China) was used to measure the microhardness of both the 04Cr13Ni5Mo base metal and composite coatings under a load of 200 gf for 10 s, with the final average value representing the microhardness measurement. The error was indicated by standard deviation, while the coating surface-to-base metal microhardness was measured. Wear tests utilized 5 mm diameter WC balls as paired materials under experimental conditions: 20 N normal load, 3 Hz frequency (repetitive motion), 10 mm sliding distance (amplitude ± 5 mm), and a test duration of 30 min. Post-wear analysis involved scanning electron microscopy (SEM) combined with energy dispersive X-ray spectroscopy (EDS) to examine microstructural and compositional characteristics, thereby elucidating wear mechanisms and friction chemistry interactions. The morphology and depth profile of the wear track were characterized using a 3D optical profilometer to quantify the wear volume and assess the surface damage. Following mirror-polishing treatment, the samples were placed in a cavitation test machine (XO-1200, 50 μm, Xian’ou Nanjing, China) for ultrasonic cavitation tests in deionized water (20 ± 2 °C). The distance between the cavitation probe and the sample was maintained at 1 mm, and the cavitation characteristics were summarized by measuring the weight loss of the coating at different time intervals.

## 3. Results and Discussion

### 3.1. Microscopic Morphology and Mechanical Properties

**Figure 1:**
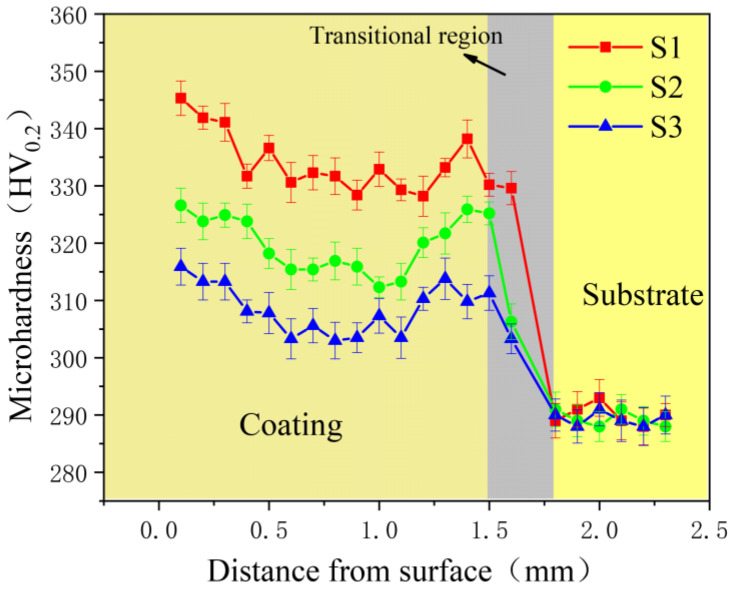
Hardness distribution across S1, S2, and S3 coatings and base material.

Figure 1 shows the microhardness distribution of alloy coatings prepared with different laser powers. It is evident that all coatings exhibit significantly higher microhardness than the base material, with the hardness progressively decreasing as laser power increases. Sample S1 demonstrates higher microhardness than samples S2 and S3, with the average microhardness values for the three coatings being 345.3HV_0.2_, 326.6HV_0.2_, and 307.5HV_0.2_, respectively. Analysis revealed that the BCC phase in S1 coating was slightly higher than in S2 and S3. This phenomenon occurs because increased laser power induces a phase transformation during laser cladding, generating trace amounts of hard phases that enhance material hardness. The experimental results demonstrate that reducing laser power appropriately yields higher hardness than increasing it. This is because thermal input and cooling rate are closely related during laser cladding. Lower laser power primarily reduces matrix dilution, which allows for optimal molten pool temperature and cooling rate. The decreased cooling rate further promotes grain growth, ultimately resulting in enhanced material hardness. The microstructure and XRD analysis revealed that some hard phases precipitated in the BCC phase samples, while the grain size was reduced in the under-eaten microstructure. This indicates that the primary mechanism in the BCC system is grain refinement, whereas the FCC system mainly involves lattice distortion, resulting in localized chemical order in the grains [12].

**Figure 2:**
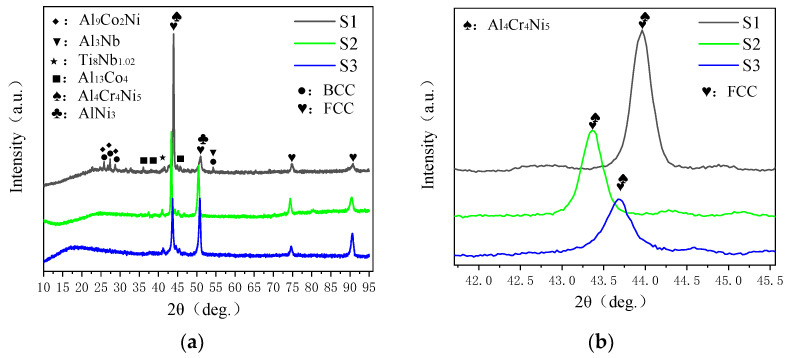
(**a**) XRD patterns of Co_37.4_Cr_30_Ni_20_Al_5_Ti_5_Nb_2.6_ alloy coatings. (**b**) Partial enlarged drawing.

Figure 2a displays the XRD patterns of the sample, which primarily exhibits a face-centered cubic (FCC) phase microstructure. When laser power is appropriately adjusted, BCC phase is generated while the relative content of FCC phases decreases. Notably, the peak positions in XRD patterns at 3200 W and 3400 W laser powers remain largely consistent. At 3000 W laser power, intermetallic compounds of Al, Co, and Ni are formed, typically exhibiting complex crystal structures and high hardness, leading to the generation of hard phases. Additionally, under the higher laser power of 3400 W, the molten pool demonstrates improved fluidity. This may result in more uniform dissolution of Nb elements in the melt or formation of other more stable phases, thereby reducing the number of Nb atoms required to form Al3Nb. At 3000 W power, the reduced thermal input may decrease Nb’s overall solubility in the melt or minimize dilution effects from matrix elements on the cladding layer, enabling more efficient bonding between Nb and Al to form the Al3Nb phase. The Al-Nb Laves phase or similar intermetallic compounds exhibit exceptional hardness and stability. Additionally, within the laser power range studied, TiNb phases predominantly form at grain boundaries, typically existing in the BCC structure. These TiNb phases act as crack stoppers at grain boundaries and slow down cavitation-induced crack propagation. This mechanism explains why all three test samples demonstrated significantly better cavitation performance compared to the matrix material. As laser power increases, the molten pool temperature rises, causing the AlCrNi phase to decompose or dissolve at high temperatures, thereby suppressing nucleation. Additionally, the extended cooling time allows sufficient time for phase transformation and growth. The AlNi phase consumes substantial amounts of Al and Ni, resulting in a melt composition unsuitable for AlCrNi phase formation. Consequently, XRD analysis reveals a significant enhancement of AlNi phase diffraction peaks while the AlCrNi phase peaks diminish [13].

As shown in Figure 2b, the XRD zoom images of three coatings reveal that in the laser-clad Co_37.4_Cr_30_Ni_20_Al_5_Ti_5_Nb_2.6_ high-entropy alloy coating, when the laser power was 3000 W, the FCC main peak showed a rightward shift compared to 3200 W and 3400 W. This typically indicates a reduction in the lattice constant of the FCC phase. Such phenomena are closely related to changes in thermal input, cooling rates, element dissolution behavior, and potential internal stress states caused by variations in laser power. When the laser power is 3000 W, the hard phases containing Co, Al, Ni, and other elements are more likely to be precipitated from the FCC phase, which reduces the concentration of solute atoms in the FCC matrix, resulting in the reduction in lattice constant of the FCC phase and a right shift of the main peak. However, the relative content of the whole bimodal peaks does not change [14].

**Figure 3:**
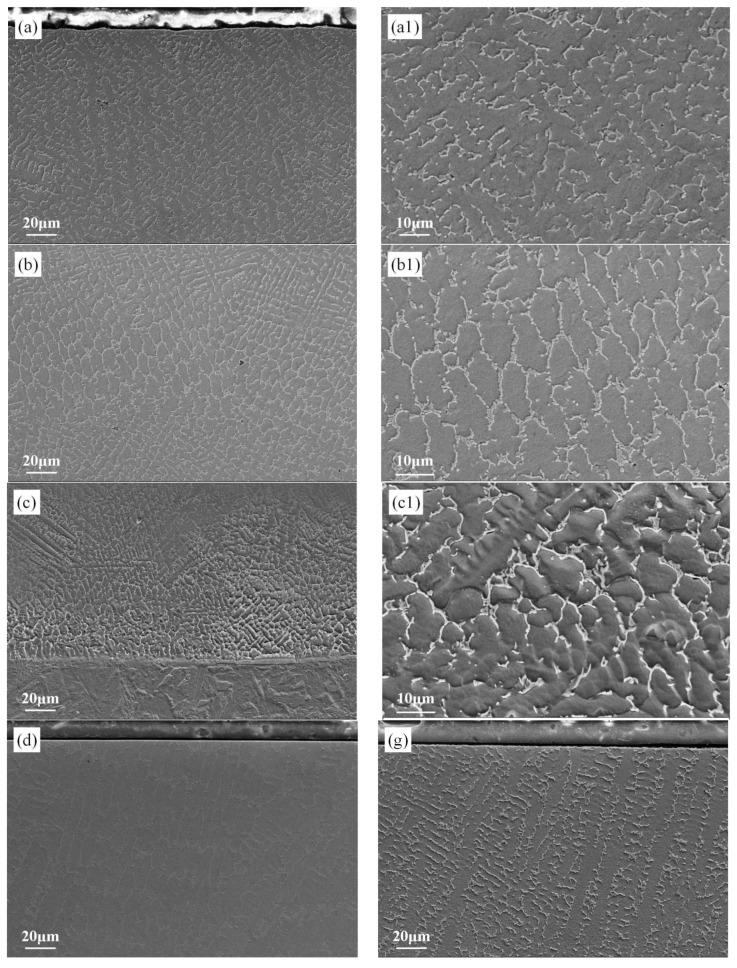

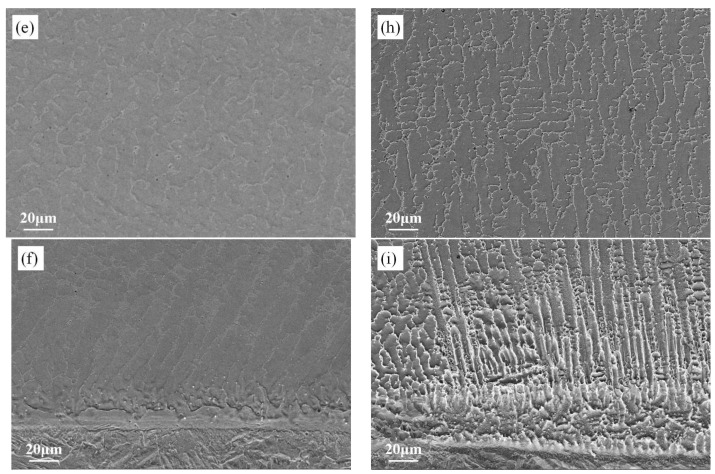
Microstructure of laser cladding alloy coating: (**a**–**c**) Microscopic morphology of upper, middle, and lower parts of S1 coating. (**a1**–**c1**) An enlarged picture of the corresponding part. (**d**–**f**) Microscopic morphology of upper, middle, and lower parts of S2 coating. (**g**–**i**) Microscopic morphology of upper, middle, and lower parts of S3 coating.

Laser cladding technology has the characteristics of fast cooling and heating, and the laser power in the process parameter directly controls the heat input. The appropriate heat input is the key to the preparation of good performance coating [15,16]. By controlling the molten pool residence time and cooling rate, the final phase composition and microstructure are determined. Figure 3 presents microscopic images of upper, middle, and lower sections of coating samples prepared under three laser power settings (S1, S2, S3), with particular focus on the high-performance S1 coating. The experiments revealed clearly visible grain boundaries without defects and excellent density. Figure 3a–c show the microstructure of the high-entropy alloy coating fabricated at 3000 W laser power, with Figure 3a1–c1 show magnified images corresponding to upper, middle, and lower sections, respectively. The cross-sectional characteristics show that dendritic, columnar, and equiaxed crystals are distributed in the whole coating, among which the equiaxed crystals account for the largest proportion. Their distribution significantly enhances both strength and hardness. This explains why the S1 coating exhibits higher hardness than the other two samples. Furthermore, Figure 3a–c reveal that the coating’s upper and lower sections exhibit relatively finer grains, while the central section shows larger grains. This variation primarily stems from faster heat dissipation in the upper/lower sections where grain growth duration is shorter, preventing substantial size expansion. Conversely, the central section experiences slower heat dissipation, allowing longer grain growth periods and consequently larger grain sizes. This mechanism explains why the microhardness of the coating’s upper/lower sections is higher than that of the central region. This phenomenon also exists in S2 and S3 [17].

Moreover, the distribution of these crystals in laser cladding coatings substantially affects cavitation performance, particularly through the initiation and propagation of cavitation cracks. These findings confirm that Sample S1 demonstrates superior cavitation resistance compared to the other two test groups. The formation of equiaxed grain structures in the coating prevents severe local segregation, thereby eliminating large-scale peeling issues caused by compositional inhomogeneity. The tortuous grain boundaries effectively deflect, branch, and impede microcrack propagation. For cracks to penetrate the equiaxed structure, they must expend more energy, resulting in longer and more complex growth paths. This significantly enhances the material’s fatigue life and cavitation resistance. The fine-grained equiaxed structure not only provides high strength and hardness through grain refinement to withstand initial indentation, but also exhibits excellent toughness due to abundant grain boundaries that facilitate coordinated plastic deformation. This combination of strength and toughness makes it an ideal choice for resisting cavitation, a fatigue process. Figure 3d–f and Figure 3g–i, respectively, demonstrate the microstructural morphology of the S3 sample’s cross-section in the neutralized lower region and the S2 sample’s corresponding section. The unique microstructure evolution stems directly from the laser cladding process: temperature gradients and controlled solidification rates induce columnar crystals at the base, equiaxed crystals in the middle zone, and dendritic structures at the tip. Experimental observations reveal that all specimens exhibit columnar dendrites at the apex. During cavitation, these microcracks develop continuous straight paths with reduced propagation resistance, but their rapid downward propagation along vertical crystal boundaries significantly diminishes cavitation performance. The microstructures of S2 and S3 exhibit columnar crystal-branching dendrites arranged from bottom to top. While S1 samples also display branching structures in their coating center, they contain small amounts of equiaxed crystals [18]. This distinctive microstructure demonstrates significantly superior cavitation resistance compared to the base material. Experimental results confirm that when laser power reaches 3000 W, the coating achieves optimal cavitation resistance through its unique three-dimensional structure comprising columnar dendrites, equiaxed crystals, and subsequent dendrites.

**Figure 4:**
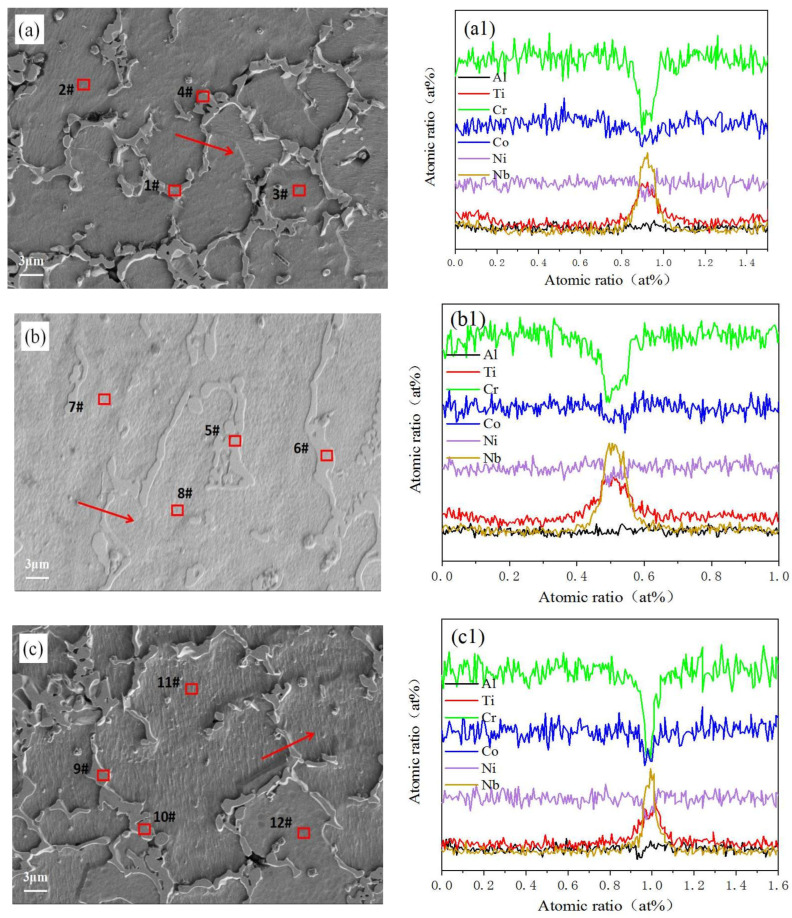
Distribution of elements at and around the coating grain boundary: Panels (**a**–**c**) show the microstructures at S1–S3 scanning positions, respectively. (**a1**–**c1**) Line scan test results at corresponding positions of S1–S3.

Figure 4a–c present magnified microstructural images of three samples at 2000× scale, with spot scanning and line scanning analyses conducted at designated locations during testing. Table 2 shows elemental distribution at corresponding points, while Figure 4a1–c1 display line scan patterns at these positions. Notably, grain boundaries exhibit a Cr-deficient phase enriched with Nb and Ti phases. XRD analysis confirms the formation of TiNb phases at these interfaces, demonstrating that this unique phase structure can effectively inhibit the crack propagation caused by cavitation [19]. This validates the coating’s superior cavitation resistance achieved through the experimental formulation. The S1 sample’s equiaxed crystal structure combined with the TiNb phase exhibited enhanced crack propagation inhibition properties, outperforming both S2 and S3 samples in cavitation resistance. Furthermore, elemental analysis of corresponding points revealed that the Nb and Ti atoms in S1 samples exhibited higher concentration at grain boundaries compared to S2 and S3 samples. A significant negative mixing enthalpy was observed between the excess Nb and Al atoms. This indicates that Nb and Al exhibit energy-driven mutual attraction, tending to combine and form compounds such as the AlNb phase. At the same time, the analysis of the line scan diagram showed that the decreasing trend of the Co atom content in S1 sample was lower than that of the other two groups of samples. At the appropriate laser power of 3000 W, some Co elements aggregated and formed a new phase with Al and Ni elements, which played a unique role in resisting cavitation [20].

### 3.2. Friction Wear Performance

**Figure 5:**
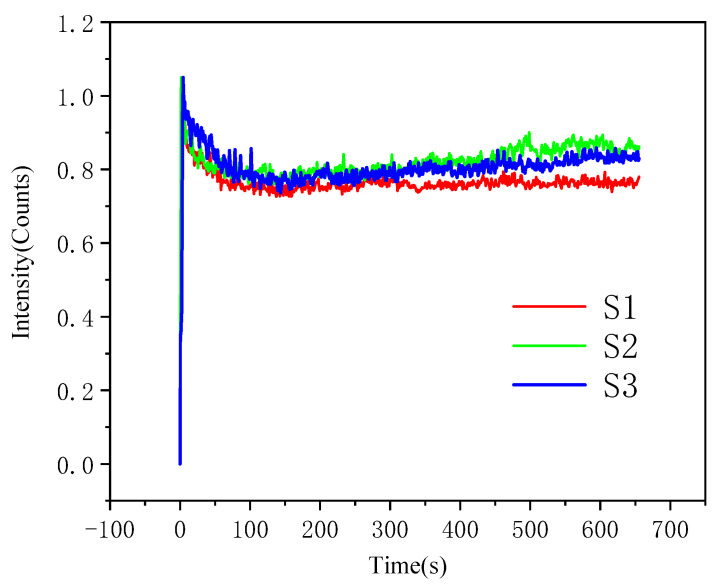
Friction wear curve of laser cladding alloy coating.

Figure 5 shows the wear-time-dependent coefficient of friction curves for alloy coatings prepared with different laser powers. The S1 sample exhibited lower coefficients compared to other specimens [21,22]. During the initial wear test phase, a brief dip in friction coefficient was observed, primarily attributed to the lubricating friction film formed between the sample surface and the friction pair, which effectively reduced interfacial shear strength. As the test progressed, the actual contact area between the friction pair and the sample gradually increased, leading to a steady rise in friction coefficient until reaching a dynamic equilibrium state. According to the relationship between material friction coefficients and wear resistance, S1 demonstrated optimal wear performance with the lowest coefficient and weight loss values.

**Figure 6:**
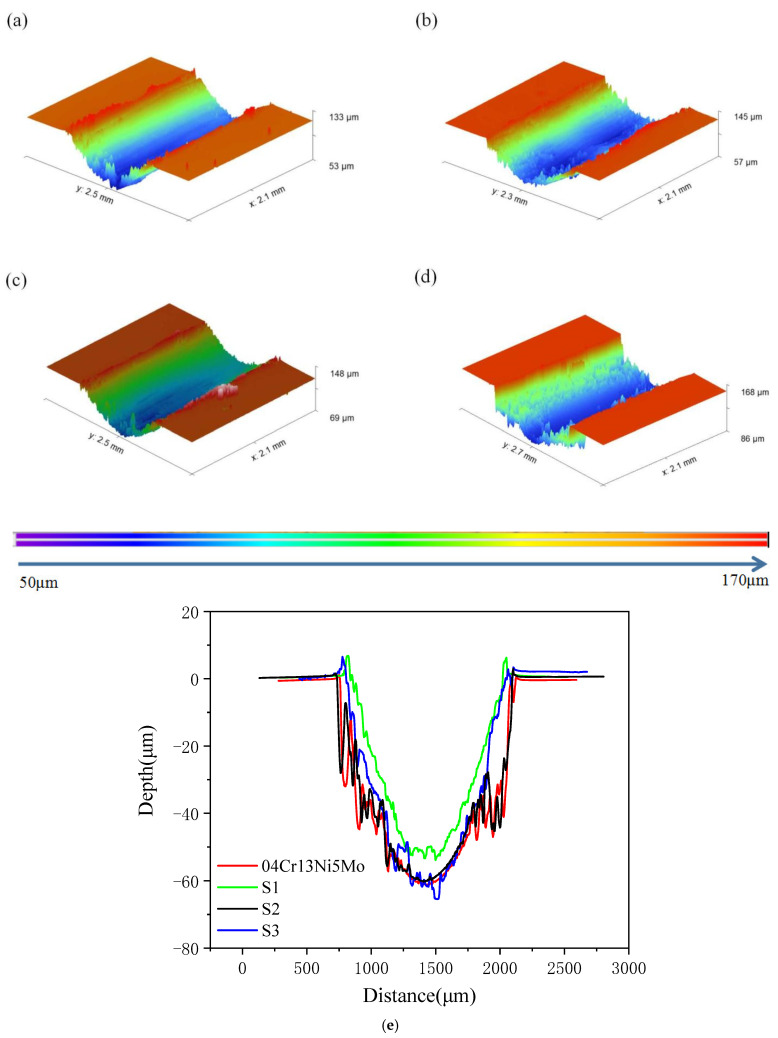
3D surface topography and 2D cross-section profile of alloy coatings after wear under 20 N load: (**a**) S1, (**b**) S2, (**c**) S3, (**d**) 04Cr13Ni5Mo. (**e**) 2d cross-section profile of wear marks.

Sample S1 demonstrated superior wear resistance compared to the other two groups. The friction coefficient and weight loss of S2 and S3 were larger than that of S1, which may be due to the partial decomposition of the hard phase under the action of high laser power, resulting in the softening of the coating, leading to the increase in their friction coefficient and the reduction in wear resistance. For the S1 coating, a hard phase was formed by the grain boundary anchoring effect and microhardening during the rapid cooling process, which improved the wear resistance of the coating. Figure 6 shows the two-dimensional and three-dimensional wear morphology at room temperature. Consistent with the friction direction, all coatings developed uneven plow groove marks. S1 exhibited the smallest wear depth within the same measurement distance, indicating superior wear resistance. The wear rate calculation formula for coatings is Wt = Vloss/FNL, where Wt represents wear rate, Vloss denotes the worn volume, F_N_ stands for load, and L indicates total sliding distance. Table 3 presents the wear rates of three coatings under a 20 N force. After 30 min of wear, the wear rate increased from 1.68 × 10^−4^ mm^3^/N·m to 1.68 × 10^−4^ mm^3^/N·m, demonstrating that Sample S1 exhibited a 16.07% improvement in wear resistance compared to the matrix material [23].

**Figure 7:**
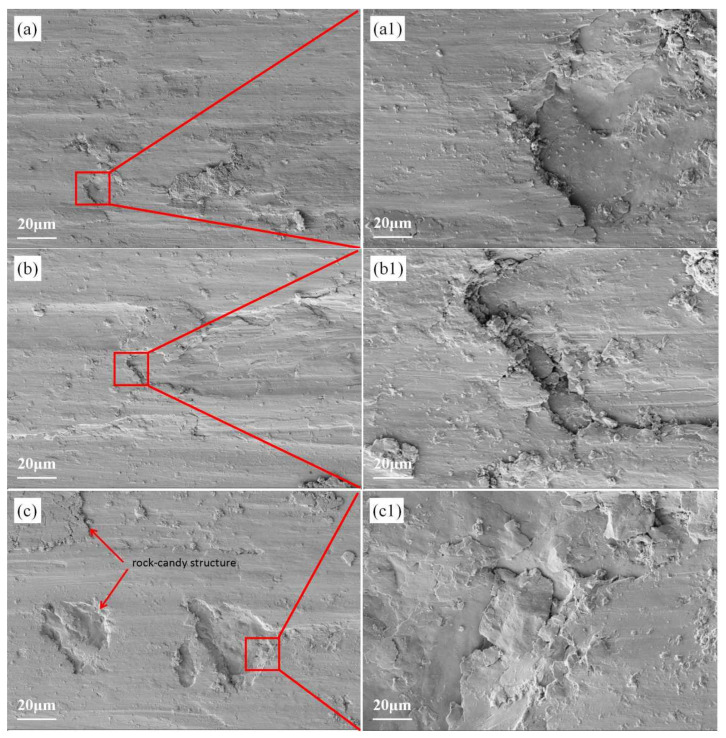
(**a**) Microscopic morphology of S1 coating after wear. (**b**) Microscopic morphology of S2 after wear. (**c**) Microscopic morphology of S3 after wear. (**a1**–**c1**) Local magnified micrographs of coating after wear.

Figure 7 shows the surface wear morphology of three coatings under 20 N load after 30 min of wear at room temperature. All three coatings developed distinct plow groove patterns along the friction direction. Figure 7a,a1 display the microstructure of the 3000 W laser power sample post-wear. As shown in Figure 7a, the S1 coating surface showed no visible cracks after wear, only microcutting marks forming plow groove-like patterns, indicating typical abrasive wear mechanisms. With time, the coating delamination intensified, releasing more abrasive particles. Figure 7b–c1 reveal the microstructures and magnified views of S2 and S3 post-wear. The experiments demonstrated deeper fracture surfaces and increased abrasive particles following brittle fracture material spalling.

**Figure 8:**
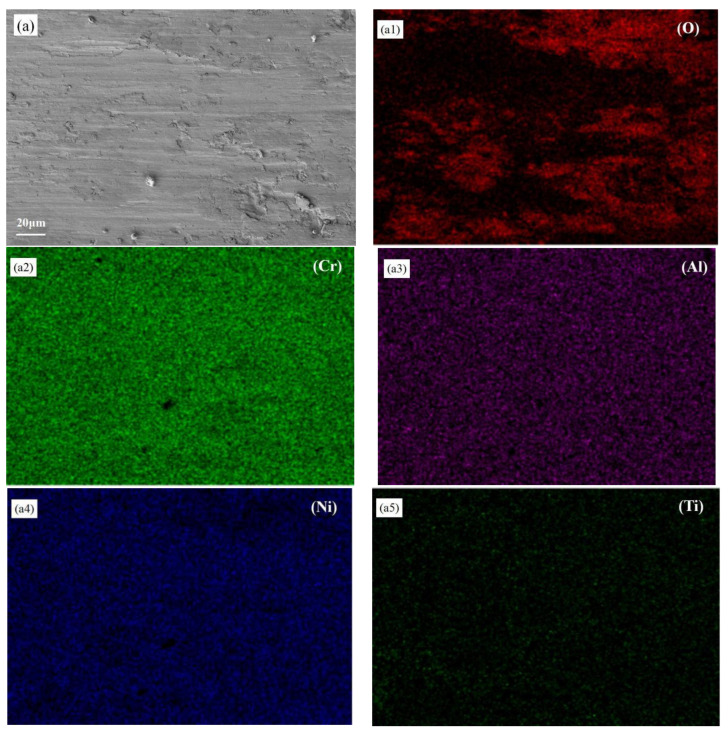

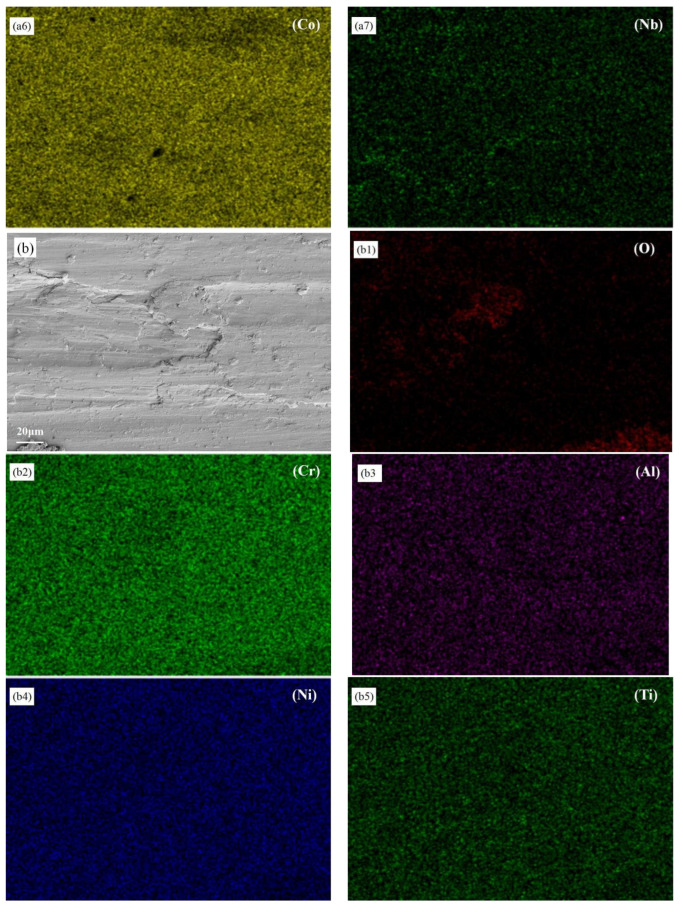

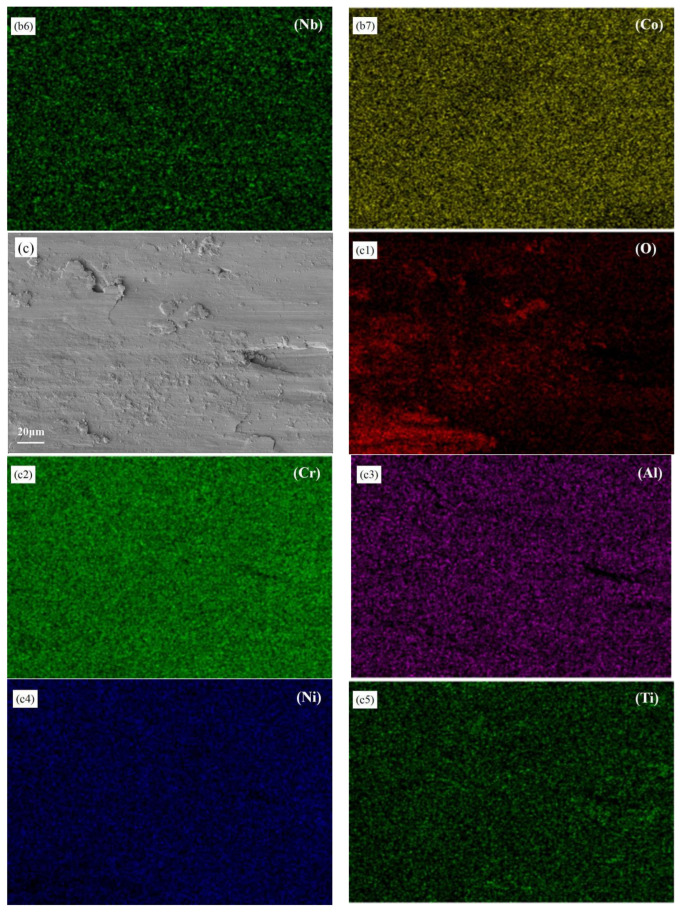

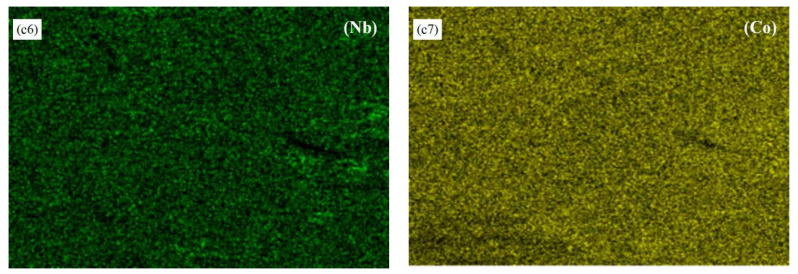
Surface composition distribution after wear of laser cladding alloy coating under 20 N load: (**a**) S1, (**b**) S2, (**c**) S3. (**a1**–**a7**), (**b1**–**b7**), and (**c1**–**c7**) are the distribution charts of the corresponding elements, respectively.

Figure 8 illustrates the elemental distribution after wear of three coatings. The analysis reveals a relatively uniform elemental distribution within the coatings, all containing oxygen that forms localized oxide layers on the surface. This phenomenon primarily occurs due to heat generated by reciprocating friction, which induces oxidation on the surface, forming an oxide layer that adheres to the worn areas and causes localized oxidative wear. Comparing Figure 8a1,b1,c1, it can be found that the oxygen content distribution shown in Figure 8b1 is lower, while the corresponding S2 sample shows the highest degree of wear. This may be attributed to the absence of continuous, dense, and stable protective oxide film during the wear process. This results in wear mechanisms dominated by abrasive wear or fatigue wear, leading to accelerated material loss. Additionally, phase composition analysis via XRD revealed that S1 samples exhibited higher proportion of hard phases compared to S2 and S3 samples. An appropriate proportion of hard phases can significantly enhance the material’s resistance to scratching and plowing, thereby improving the coating’s wear resistance.

### 3.3. Cavitation Erosion Performance

**Figure 9:**
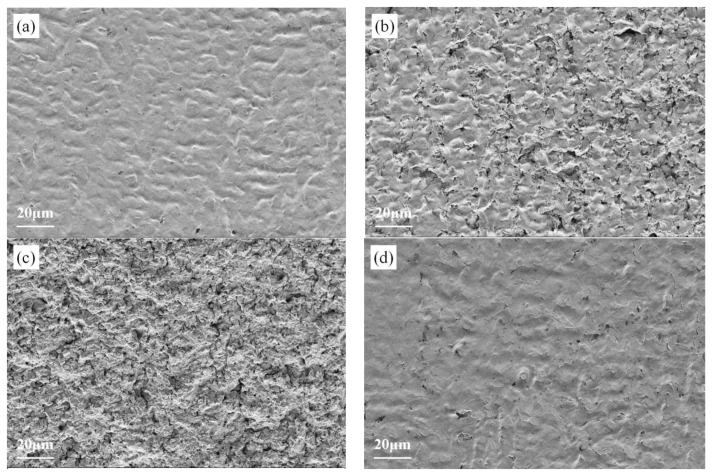

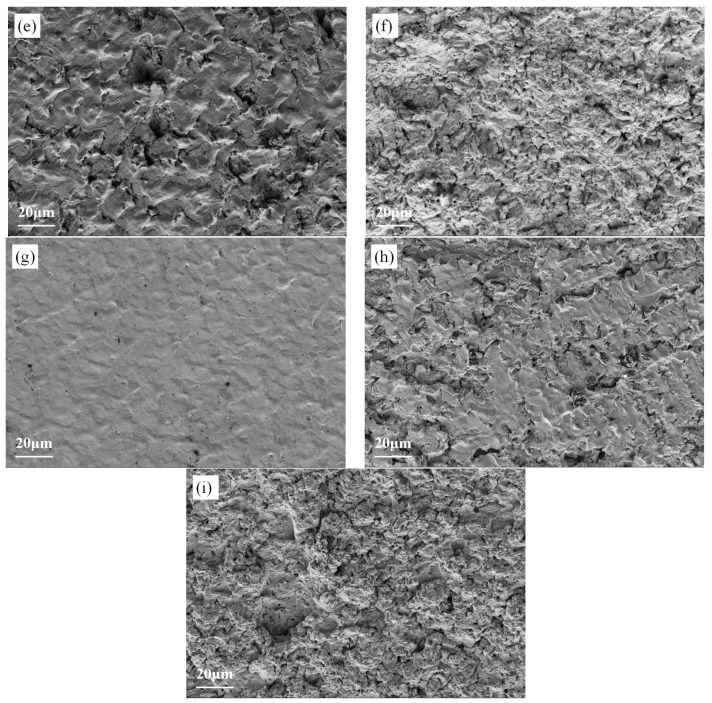
The surface microstructure of laser cladding alloy coatings after cavitation erosion: (**a**) S1 cavitation after 1 h, (**b**) S1 cavitation after 4 h, (**c**) S1 cavitation after 20 h, (**d**) S2 cavitation after 1 h, (**e**) S2 cavitation after 4 h, (**f**) S2 cavitation after 20 h, (**g**) S3 cavitation after 1 h, (**h**) S3 cavitation after 4 h, (**i**) S3 cavitation after 20 h.

Figure 9 displays the microstructural evolution of three coatings during cavitation damage progression. In the initial three-hour pre-cavitation phase (Figure 9a,d,g), no material loss was observed in the specimens. Notably, Sample S1 exhibited smaller damaged regions during this phase compared to the other two groups, demonstrating enhanced cavitation resistance. The optimal cavitation performance achieved when laser power was reduced to 3000 W likely resulted from grain refinement and formation of favorable microstructures that minimized structural defects. Furthermore, observing the cavitation stabilization phases of the three sample groups, all specimens exhibited full-area surface spalling. Under the same magnification as shown in Figure 9c,f,i, S1 specimens displayed smaller and more densely distributed cavitation pits. This finding is highly consistent with the earlier observation of Sample S1’s smaller cavitation acceleration zone area. The denser and smaller cavities effectively disperse cavitation damage across the entire surface rather than concentrating it in a few weak points. This enhances the coating’s cavitation resistance, prolonging the stabilization phase and resulting in minimized mass loss. Additionally, the power-optimized multiphase structure demonstrates superior cavitation resistance, manifesting as optimized pit morphology at the microscale and enhanced cavitation resistance at the macroscale [24].

**Figure 10:**
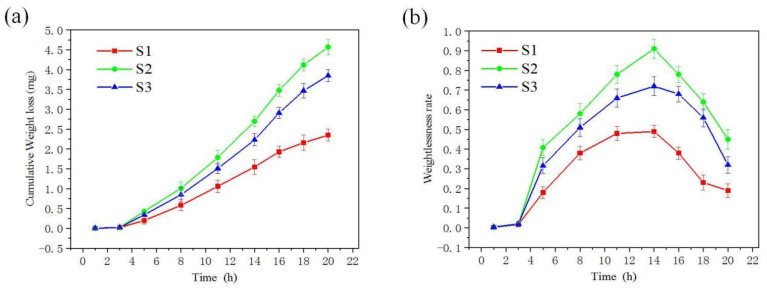
Weight loss curve of laser cladding alloy coating under cavitation erosion with time: (**a**) weight loss at different times, (**b**) weight loss rate at different times.

Figure 10 illustrates the weight loss patterns of samples prepared at three power levels under cavitation effects over time. As shown in Figure 10a, no coating exhibited significant weight loss during the initial 2 h cavitation phase, indicating that during the latent stage of cavitation, the material effectively buffered external microjet impacts through its inherent plastic deformation capacity, thereby delaying surface damage progression. With extended cavitation duration, all coatings experienced progressively increasing weight loss. Figure 10b shows that after the cavitation latent period, all coatings began to experience weight loss. The weight loss rate initially rose rapidly, then gradually slowed down, eventually declining and stabilizing. As shown in Figure 10a, Sample S1 demonstrated the best cavitation resistance, losing only 2.35 mg of mass after 20 h of cavitation. In contrast, Samples S2 and S3 lost 4.57 mg and 3.85 mg of mass, respectively, after the same duration. The conventional turbine stainless steel 04Cr13Ni5Mo material showed a weight loss of 21.15 mg under identical cavitation conditions. All three coatings exhibited significantly superior cavitation resistance compared to the base material.

## 4. Conclusions

(1) Effect of different laser powers on hardness: With the increase in laser power, the microhardness of the coating decreases because the strengthened phase in the coating is dissolved due to the increase in thermal input energy.

(2) The best wear resistance was achieved at 3000 W laser power. A 30 min dry sliding wear test under 20 N load revealed that the gradual increase in alloy coating hardness and the formation of trace hard phases effectively enhances wear performance. Data showed the wear rate decreased from 1.63 × 10^−4^ mm^3^/(N·m) at 3200 W to 1.41 × 10^−4^ mm^3^/(N·m) at 3000 W. Compared with the base material (04Cr13Ni5Mo), the coating’s wear resistance increased by 16.07% at 3000 W laser power.

(3) The coating exhibited exceptional cavitation resistance at 3000 W laser power. At this power, the best cavitation resistance was achieved. At 3000 W, the coating demonstrated optimal performance, showing only 2.35 mg of mass loss during a 20 h ultrasonic cavitation test. In contrast, the conventional turbine stainless steel 04Cr13Ni5Mo under identical conditions exhibited 21.15 mg of mass loss. This indicates that Co_37.4_Cr_30_Ni_20_Al_5_Ti_5_Nb_2.6_ high-entropy alloy coatings prepared with appropriate laser power achieve a qualitative leap in cavitation resistance.

## Figures and Tables

**Table 1:** Process parameters.

Sample Number	Laser Power (W)	Scanning Speed (mm/s)	Powder Feed Rate (g/min)
S1	3000	7	32
S2	3200	7	32
S3	3400	7	32

**Table 2:** Element composition distribution of corresponding points (at%).

Element/Number	1#	2#	3#	4#	5#	6#	7#	8#	9#	10#	11#	12#
Al	3.91%	4.86%	3.87%	3.91%	3.27%	3.05%	3.98%	3.78%	3.04%	3.66%	3.48%	3.96%
Ti	9.20%	2.13%	2.65%	10.02%	10.36%	9.09%	3.87%	3.32%	12.70%	10.05%	2.96%	3.10%
Cr	24.09%	37.56%	36.44%	23.18%	25.03%	27.21%	36.40%	37.49%	22.63%	24.35%	37.09%	37.38%
Co	31.77%	35.10%	35.64%	30.80%	30.90%	30.86%	33.34%	34.22%	30.89%	31.19%	35.48%	34.01%
Ni	15.68%	19.82%	20.48%	15.86%	16.57%	16.83%	20.75%	19.86%	15.60%	17.67%	19.93%	20.49%
Nb	15.35%	0.52%	0.92%	16.22%	13.87%	12.9%6	1.66%	1.33%	15.14%	13.08%	1.07%	1.06%

**Table 3:** Wear rate of matrix and coatings under different process conditions.

Sample/Load (N)	20 N
S1	1.41 × 10^−4^ mm^3^/N·m
S2	1.63 × 10^−4^ mm^3^/N·m
S3	1.49 × 10^−4^ mm^3^/N·m
04Cr13Ni5Mo	1.68 × 10^−4^ mm^3^/N·m

## Data Availability

The original contributions presented in this study are included in the article. Further inquiries can be directed to the corresponding authors.
